# Dynamic changes of rumen bacteria and their fermentative ability in high-producing dairy cows during the late perinatal period

**DOI:** 10.3389/fmicb.2023.1269123

**Published:** 2023-09-25

**Authors:** Yongxia Mao, Feifei Wang, Weiyi Kong, Ruiling Wang, Xin Liu, Hui Ding, Yun Ma, Yansheng Guo

**Affiliations:** ^1^College of Animal Science and Technology, Ningxia University, Yinchuan, China; ^2^Key Laboratory of Ruminant Molecular and Cellular Breeding of Ningxia Hui Autonomous Region, College of Animal Science and Technology, Ningxia University, Yinchuan, China

**Keywords:** high-producing dairy cow, metabolic challenges, rumen bacteria, ruminal fermentation capacity, late perinatal period

## Abstract

**Background:**

High-producing dairy cows face varying degrees of metabolic stress and challenges during the late perinatal period, resulting in ruminal bacteria abundance and their fermentative ability occurring as a series of changes. However, the dynamic changes are still not clear.

**Aims/methods:**

Ten healthy, high-producing Holstein dairy cows with similar body conditions and the same parity were selected, and ruminal fluid from the dairy cows at postpartum 0, 7, 14, and 21 d was collected before morning feeding. 16S rRNA high-throughput sequencing, GC-MS/MS targeted metabolomics, and UPLC-MS/MS untargeted metabolomics were applied in the study to investigate the dynamic changes within 21 d postpartum.

**Results:**

The results displayed that the structures of ruminal bacteria were significantly altered from 0 to 7 d postpartum (*R* = 0.486, *P* = 0.002), reflecting the significantly declining abundances of Euryarchaeota and Chloroflexi phyla and *Christensenellaceae, Methanobrevibacter*, and *Flexilinea* genera (*P* < 0.05) and the obviously ascending abundances of *Ruminococcaceae, Moryella, Pseudobutyrivibrio*, and *Prevotellaceae* genera at 7 d postpartum (*P* < 0.05). The structures of ruminal bacteria also varied significantly from 7 to 14 d postpartum (*R* = 0.125, *P* = 0.022), reflecting the reducing abundances of *Christensenellaceae, Ruminococcaceae*, and *Moryella* genera (*P* < 0.05), and the elevating abundances of *Sharpea* and *Olsenella* genera at 14 d postpartum (*P* < 0.05). The metabolic profiles of ruminal SCFAs were obviously varied from 0 to 7 d postpartum, resulting in higher levels of propionic acid, butyric acid, and valeric acid at 7 d postpartum (*P* < 0.05); the metabolic profiles of other ruminal metabolites were significantly shifted from 0 to 7 d postpartum, with 27 significantly elevated metabolites and 35 apparently reduced metabolites (*P* < 0.05). The correlation analysis indicated that propionic acid was positively correlated with *Prevotellaceae* and *Ruminococcaceae* (*P* < 0.05), negatively correlated with *Methanobrevibacter* (*P* < 0.01); butyric acid was positively associated with *Prevotellaceae, Ruminococcaceae*, and *Pseudobutyrivibrio* (*P* < 0.05), negatively associated with *Christensenellaceae* (*P* < 0.01); valeric acid was positively linked with *Prevotellaceae* and *Ruminococcaceae* (*P* < 0.05); pyridoxal was positively correlated with *Flexilinea* and *Methanobrevibacter* (*P* < 0.05) and negatively correlated with *Ruminococcaceae* (*P* < 0.01); tyramine was negatively linked with *Ruminococcaceae* (*P* < 0.01).

**Conclusion:**

The findings contribute to the decision of nutritional management and prevention of metabolic diseases in high-producing dairy cows during the late perinatal period.

## 1. Introduction

In recent decades, improvements in breeding and nutritional technology have contributed to a sustained increase in milk production to meet the human demand for milk (Capper et al., 2009). Milk production has almost doubled in many countries around the world in the last 30 years (von Keyserlingk et al., 2013). However, the continued growth in milk production poses a serious challenge to the metabolism and health of dairy cows, especially in the late perinatal period (within 21 d after parturition) (Trevisi et al., 2012; Gross and Bruckmaier, 2019). When lactation initiates, dairy cows preferentially deliver nutrients to the mammary gland to supply energy requirements for lactation, which means the body requires higher energy and nutrient requirements than the dry period (Oftedal, 2011). Hence, dairy cows are prone to a physiological state of negative energy balance (NEB) after parturition due to lactation initiation and reduced dry matter intake (DMI) (Vossebeld et al., 2022). Although complex adaptation processes enable dairy cows to maintain the homeostasis of energy and nutrients, many individuals, especially high-producing individuals, fail to successfully cope with NEB (van Knegsel et al., 2013). High-producing cows experience varying degrees of metabolic stress at calving (LeBlanc, 2010). The metabolic challenges can affect the immune, metabolic, and endocrine systems, resulting in disorders in hormone, glucose, and lipid metabolisms of high-producing dairy cows during the late perinatal period (Esposito et al., 2014). The concentrations of many metabolic hormones and their receptors can change under the influence of parturition (Lucy et al., 2001). Leptin is a type of peptide hormone secreted by adipose tissue that can influence voluntary feeding in dairy cows (Ingvartsen and Boisclair, 2001). Low concentrations of leptin after parturition can lead to less DMI intake, proceeding to impact the fermentative ability of rumen bacteria in dairy cows (Wathes et al., 2007).

Ruminal bacteria participate in the digestion and nutrient absorption of ruminants (Pinnell et al., 2022), which ferment fiber in feed as short-chain fatty acids (SCFAs) to provide ~70% of energy for dairy cows (Indugu et al., 2017). The rumen bacteria tend to fluctuate due to the diet, environment, and physiological status (Bharanidharan et al., 2021). Lactation initiation and NEB result in obvious fluctuation in the abundance of rumen bacteria and concentrations of fermentation products in dairy cows (Pitta et al., 2014). Our previous study found that the abundance of rumen bacteria was significantly lower in dairy cows after parturition when compared with before parturition (Guo et al., 2023). The changes in ruminal bacterial composition after parturition can lead to some alterations in rumen metabolism (Plaizier et al., 2008; Auffret et al., 2017). A significant correlation is observed between SCFAs and bacteria abundances in the rumen (Liu et al., 2022). Some metabolites are associated with bacteria in the rumen (Fozia et al., 2013). Therefore, due to the parturition, lactation initiation, feed change, and adaptations, the changes in rumen bacteria abundance and their fermentative ability in high-producing dairy cows within 21 d after parturition are complicated and need to be further clarified.

Currently, 16S rRNA high-throughput sequencing has been successfully applied to study the structure and quantity of rumen and gut microbiota of dairy cows (Thoetkiattikul et al., 2013; Guo et al., 2015). GC-MS/MS-targeted metabolomics can be used to detect small molecules such as amino acids, lipids, and organic acids (Zhang et al., 2020) and has been widely used for the determination of metabolites in rumen fluid, blood, and urine of ruminants (Matthews et al., 2019). UPLC-MS/MS untargeted metabolomics has also been widely adopted to qualify and quantify rumen metabolites because of its high sensitivity and accuracy (Luo et al., 2019). Therefore, the combined application of the above technologies can provide a high feasibility to comprehensively reveal the dynamic changes in rumen bacteria and their fermentative ability in high-producing dairy cows within 21 d after parturition. In this study, the concentrations and correlations of ruminal bacteria, SCFAs, and other metabolites in high-producing dairy cows at 0, 7, 14, and 21 d postpartum were studied with 16S rRNA high-throughput sequencing, GC-MS/MS targeted metabolomics, and UPLC-MS/MS untargeted metabolomics, aiming to provide some references for nutritional regulation and prevention of metabolic diseases in high-producing cows during the late perinatal period.

## 2. Materials and methods

### 2.1. Collection and group of ruminal fluids

Ten healthy high-producing Holstein cows (body weight, 600 ± 20 kg; body condition score, 3.4–3.7; daily milk yield, above 35 kg; parity, 2–3) were selected from a dairy farm in Ningxia province, China. The temperature in the cowshed was between 10 and 20°C, with a relative humidity of 50–70%. The lighting time of the dairy cows was controlled for 16 h. All dairy cows were fed the same TMR diet after calving (Supplementary Table 1). Ruminal fluids were collected from the 10 dairy cows at postpartum 0, 7, 14, and 21 d before morning feeding and were grouped as A, B, C, and D, respectively. Ruminal fluids of each group were labeled as A1–A10, B1–B10, C1–C10, and D1–D10. The ruminal fluids were collected by the following method: One end of the pre-rinsed and sterilized sampler with a metal filter was put into the rumen, and then, a 50 ml syringe fixed at the other end was used to extract the rumen fluid, discarding the first tube of rumen fluid to avoid saliva contamination and saving the second tube of rumen fluid. The supernatant from the rumen fluid after filtering and centrifugation was transferred to a 1.5 ml centrifuge tube and stored at −80°C for the succedent analyses.

### 2.2. 16s rRNA high-throughput sequencing of rumen bacteria communities

The total DNA of rumen bacteria was extracted from the four groups of rumen fluids using OMEGA Soil DNA Kit (M5635-02) (Omega Bio-Tek), and the purity and concentration of the genomic DNA were evaluated using 1% agarose gel electrophoresis. DNA was diluted to 1 ng-μl^−1^ in sterile water as the template; 341F (CCTAYGGGRBGCASCAG) and 806R (GGACTACNNGGGTATCTAAT) were chosen as primers to amplify the V3–V4 highly variable region of the 16S rRNA gene of rumen bacteria in a thermocycling PCR system. Two percentage agarose gel electrophoresis and Qiagen Gel Extraction Kit (Qiagen, Hilden, Germany) were, respectively, used to verify and further purify the amplified products. DNA libraries were then constructed using TruSeq DNA PCR-Free Sample Preparation Kit (Illumina, San Diego, CA, USA) and quantified by the Qubit and Q-PCR methods before sequencing on the NovaSeq6000 platform (Illumina Inc., San Diego, CA, USA).

The valid sequences of all samples after filtering and removing chimeras of raw sequencing were clustered into operational taxonomic units (OTUs) with 97% consistency with UPARSE (v7.0.1001) software. The species annotation of OTUs was carried out with the Mothur and SILVA v132 of SSUrRNA databases. The bacterial community composition of each sample was counted at the phylum and genus levels. After homogenizing the data of each sample, alpha diversity indices including Shannon, Simpson, Chao1, and ACE were calculated using QIIME. The dilution curves of alpha diversity and principal coordinate analysis (PCoA) plots of beta diversity were plotted using R software (version 2.15.3). ANOSIM analysis based on Bray–Curtis distances was used to determine the differences in bacterial communities between the four groups, and the differential species among the four groups were visualized by the *t*-test.

### 2.3. Targeted GC-MS/MS metabolomic analysis of ruminal SCFAs

After thawing and mixing, 50 μl of the rumen fluid was taken into a 1.5 ml centrifuge tube, 100 μl of 36 % chromatographic grade phosphoric acid solution was added to fully mix and then 150 μl of chromatographic grade MTBE (methyl tert-butyl ether) solvent added to the internal standard. The mixed fluid was ultrasonically processed for approximately 5 min in an ice bath to extract SCFAs and then centrifuged at 12,000 r-min^−1^ for 10 min at 4°C. In total, 90 μl of supernatant was transferred to the injection vial and stored at −20°C for subsequent targeted GC-MS/MS metabolomics analysis.

The acquisition conditions for GC-MS/MS analysis were as follows: chromatographic column was DB-FFAP column (30 m × 0.25 mm × 0.25 μm, Merck, USA), injection volume was 2 μl, injector temperature was 200°C, column flow rate was 1.2 ml-min^−1^, and carrier gas was helium. Column temperature program was set as follows: 95°C was kept for 1 min; risen to 100°C at 25°C-min^−1^ and then to 130°C at 17°C-min^−1^ and held for 0.4 min; risen to 200°C at 25°C-min^−1^, held for 0.5 min, and then run for 3 min. The temperatures of the electron ionization source, quadrupole, and transmission line were 230, 150, and 230°C, respectively; the ionization voltage was 70 eV, the scanning mode was multiple reaction monitoring (MRM), and the solvent delay time was 3.0 min.

Qualitative analysis of SCFAs was performed based on the retention time (RT), ion-pair formation, and secondary spectrum data. Quantitative analysis of SCFAs was carried out with MRM of triple quadrupole mass spectrometry. After the score and integral correction of peak areas, the standard curves and linear regression equations of SCFAs (acetic acid, propionic acid, isobutyric acid, butyric acid, isovaleric acid, valeric acid, and capric acid) were established. The concentrations of each SCFA in rumen fluid were calculated according to the linear regression equations. The obtained data of concentrations of each SCFA in rumen fluid were input into MetaboAnalyst 5.0 software, to perform targeted GC-MS/MS metabolomics analysis. Principal component analysis (PCA) of the software was applied to visualize the metabolic profiles (change trends) of ruminal SCFAs among groups, and orthogonal partial squares-discriminant analysis (OPLS-DA) of the software was used to calculate the variable importance in projection (VIP) values to classification. Univariate analysis of the software was used to calculate values of significance and fold change (FC) among the groups. The differential SCFAs among the groups were ascertained according to VIP ≥ 1, *P* < 0.05, FC ≥ 2, or FC ≤ 0.5.

### 2.4. Untargeted UPLC-MS/MS metabolomic analysis of other ruminal metabolites

The relative concentrations of other ruminal metabolites were determined by ultra-performance liquid chromatography-tandem mass spectrometry (UPLC-MS/MS). The chromatographic column was Waters ACQUITY UPLC HSS T3 C18 column (2.1 × 100 mm, 1.8 μm); mobile phase A was ultra-pure water with 0.04 % acetic acid; mobile phase B was acetonitrile with 0.04 % acetic acid; flow rate was 0.4 ml-min^−1^; column temperature was 40°C; injection volume was 2 μl. The elution gradient was set as follows: A: B was 95:5 at 0 min, 5:95 at 11 min, 5:95 at 12 min, 95:5 at 12.1 min, and 95:5 at 14 min. The electrospray ion source temperature was 500°C, ion source gas I was 55 psi, gas II was 60 psi, and gas curtain gas was 25 psi, and the mass spectrometry voltage was 5,500 V (+),−4500 V (-).

Qualitative analysis of other ruminal metabolites was performed based on retention time, ion pair information, and secondary spectral data. Quantitative analysis of other ruminal metabolites was performed using MRM of triple quadrupole mass spectrometry. After obtaining UPLC-MS/MS data from different samples, the ion chromatographic peaks of metabolites were extracted, and the peak areas of each metabolite were corrected and scored. The database containing sample numbers and peak areas was input into MetaboAnalyst 5.0 software to perform untargeted UPLC-MS/MS metabolomic analysis. The metabolic profiles of each group were analyzed by the PCA method; VIP values among the groups were calculated with the OPLS-DA method. The values of significance and FC among the groups were obtained with univariate analysis. The differential metabolites among the groups were confirmed according to VIP ≥ 1, *P* < 0.05, FC ≥ 2, or FC ≤ 0.5. Venn diagram was used to screen mutual differential metabolites among the groups.

### 2.5. Analysis of the correlation between rumen bacteria and metabolites

Spearman association analysis between the differential bacteria and metabolites was implemented with M^2^IA software (https://m2ia.met-bioinformatics.cn/). The correlation coefficient, R, is between−1 and 1, |R| > 0.4 indicates a strong correlation. *P* < 0.05 indicates that the correlation is significant; *P* < 0.01 indicates that the correlation is highly significant. The strong correlations between metabolites and bacteria were presented as network plots.

## 3. Results

### 3.1. Diversity of ruminal bacteria in dairy cows within 21 d postpartum

After OTU clustering analysis, 10,594 OTUs were obtained for the valid sequences of rumen fluid samples from the four groups. The number of OTUs in groups A, B, C, and D was 2,778, 2,583, 2,554, and 2,679, respectively. A total of 2,078 OTUs were shared among the four groups, accounting for 19.61 % of the total OTUs. The rarefaction curves of the four groups tended to be flat, indicating the number of samples was reasonable and enough to reflect the structure and quantity of ruminal bacteria in postpartum dairy cows within 21 d (Supplementary Figure 1).

**Figure 1:**
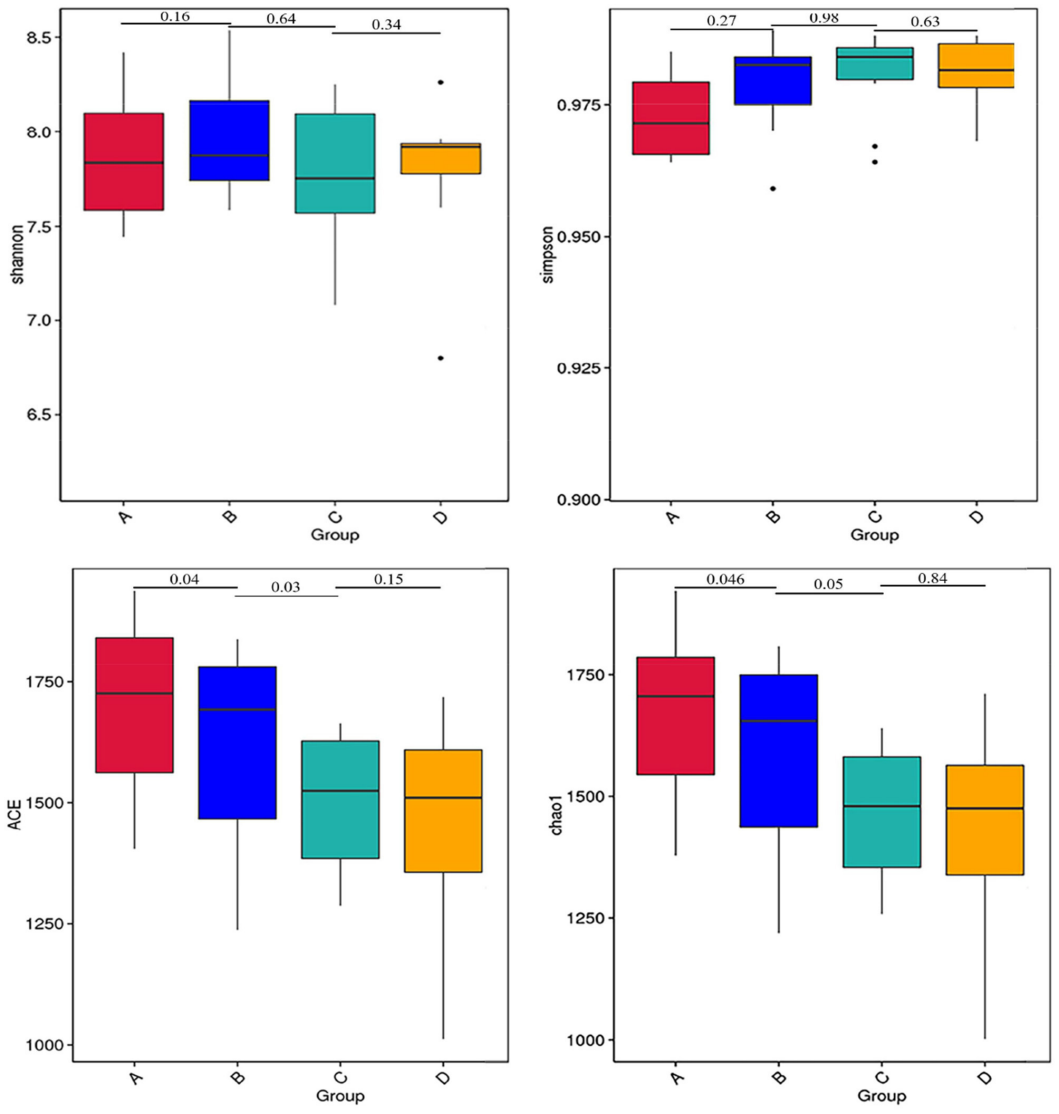
Alpha diversity indices of rumen bacteria in dairy cows within 21 d after calving. The differences observed for the alpha diversity (Shannon and Simpson indices) were not significant values (*P* > 0.05). The differences observed for the alpha diversity (ACE and Chao1 indices) between groups A and B and groups B and C were significant values (*P* < 0.05).

**Figure 2:**
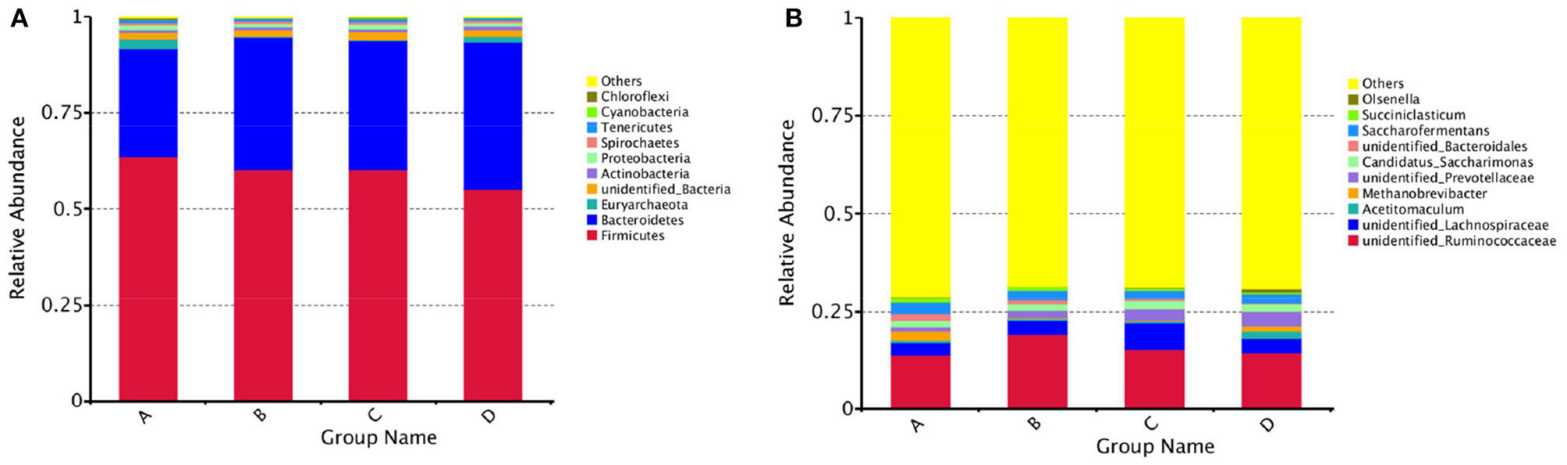
Histogram of the relative abundance of rumen species at the phylum and genus levels in dairy cows within 21 d after calving. **(A)** Bacteroidetes and Firmicutes were the dominant phyla in the rumen of dairy cows within 21 d postpartum, **(B)** unidentified_*Ruminococcaceae*, unidentified_*Prevotellaceae, Methanobrevibacter*, unidentified_*Lachnospiraceae*, and unidentified_*Bacteroidales* were the dominant genera.

The results of alpha diversity are shown in Figure 1; the Shannon and Simpson indices between groups A and B, groups B and C, and groups C and D were not significant differences (*P* > 0.05), indicating the alteration in diversity of ruminal bacteria was steady in postpartum dairy cows within 21 d. The ACE and Chao1 indices in group B were significantly lower than those in group A (*P* < 0.05) and higher than those in group C (*P* < 0.05), while there was no distinct variation between the indices of groups C and D (*P* > 0.05), suggesting that the abundance of rumen bacteria occurred as a sharp fluctuation in high-producing dairy cows within 14 d postpartum. At the phylum and genus levels, the top ten species of the four groups of rumen fluid in the relative abundance are presented in Figure 2. Firmicutes and Bacteroidetes were the dominant phyla, and unidentified_*Ruminococcaceae*, unidentified_*Prevotellaceae, Methanobrevibacter*, unidentified_*Lachnospiraceae*, and unidentified_*Bacteroidales* were the dominant genera.

**Figure 3:**
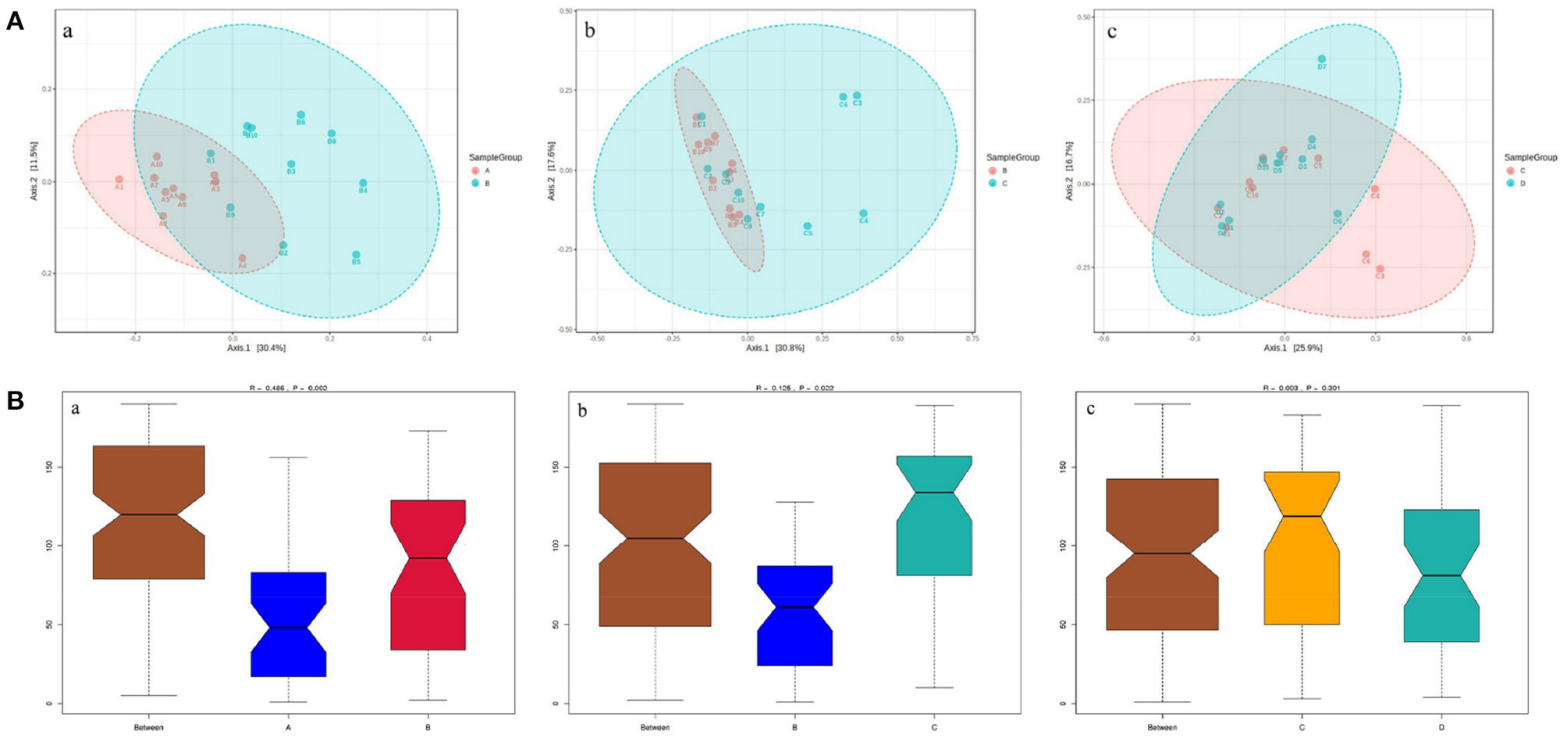
The PCoA diagrams **(A)** and boxplots **(B)** of rumen bacteria between groups A and B (a), B and C (b), and C and D (c) in dairy cows within 21 d after calving. **(A)** ANOSIM analysis of beta diversity indicated that the structure of ruminal bacteria sharply fluctuated within 14 d after parturition. **(B)** The Y-axis is the rank of the distance between the two groups, the X-axis represents intergroups; A, B, C, and D, respectively, represent their respective intragroups. R is between−1 and 1. *R* > 0 indicates significant differences between the two groups, *R* < 0, means no significant difference between the two groups.

The principal coordinate analysis (PCoA) of beta diversity analysis showed the differences in rumen bacteria structure among the four groups (Figure 3). ANOSIM analysis further revealed that there were significant differences in rumen bacteria structure between groups A and B (*R* = 0.486, *P* = 0.002) and groups B and C (*R* = 0.125, *P* = 0.022), while there was no obvious difference between groups C and D (*R* = 0.003, *P* = 0.391) (Figure 3).

### 3.2. The differential bacteria in the rumen of dairy cows within 21 d postpartum

**Figure 4:**
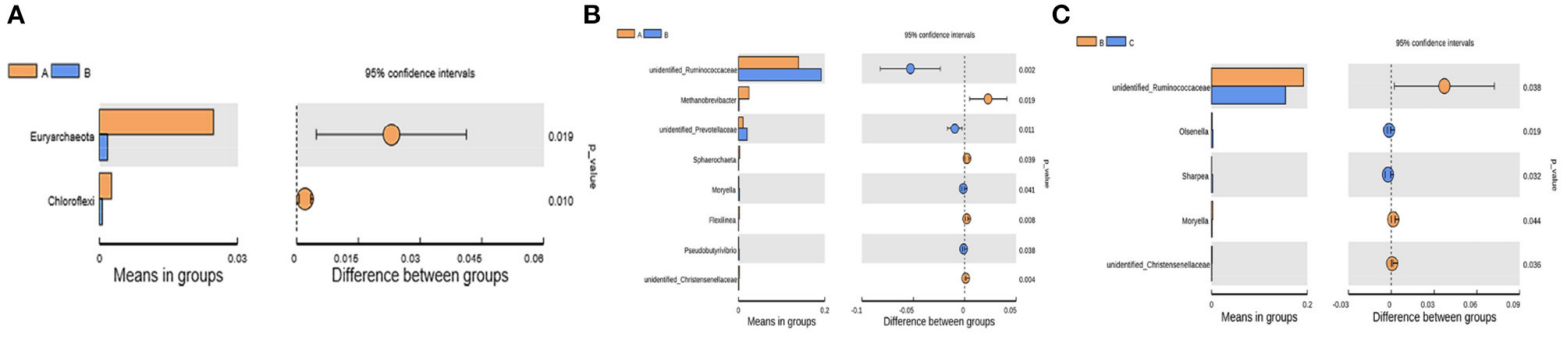
The differential rumen bacteria calculated from the *t*-test between groups A and B at the phylum and genus levels and between groups B and C at the genus level. **(A)** At the phylum level, the relative abundances of *Euryarchaeota* and *Chloroflexi* were significantly lower than those at 0 d postpartum (*p* < 0.05). **(B)** At the genus level, the relative abundances of unidentified_*Christensenellaceae, Methanobrevibacter*, and *Flexilinea* were significantly decreased within 7 d postpartum (*p* < 0.05), and unidentified_*Ruminococcaceae, Moryella, Pseudobutyrivibrio*, and unidentified_*Prevotellaceae* were significantly higher (*p* < 0.05). **(C)** The relative abundances of unidentified_*Christensenellaceae, Moryella* and unidentified_*Ruminococcaceae* significantly declined at 14 d postpartum compared with 7 d (*p* < 0.05), while those of *Sharpea* and *Olsenella* clearly increased (*p* < 0.05).

The differential bacteria at the phylum and genus levels among the four groups were obtained with the *t*-test (Figure 4). At the phylum level, the relative abundances of *Euryarchaeota* and *Chloroflexi* at 7 d postpartum were significantly lower than those at 0 d postpartum (*P* < 0.05). At the genus level, the relative abundances of unidentified_*Christensenellaceae, Methanobrevibacter* (belongs to Euryarchaeota phylum), and *Flexilinea* (belongs to Chloroflexi phylum) genera were significantly lower at 7 d postpartum than those at 0 d postpartum (*P* < 0.05). The relative abundances of unidentified_*Ruminococcaceae, Moryella, Pseudobutyrivibrio*, and unidentified_*Prevotellaceae* genera were significantly higher than those at 0 d postpartum (*P* < 0.05). Compared with 7 d postpartum, the relative abundances of *Moryella*, unidentified_*Christensenellaceae*, and unidentified_*Ruminococcaceae* genera significantly declined at 14 d postpartum (*P* < 0.05), while those of *Sharpea* and *Olsenella* clearly ascended (*P* < 0.05). No obviously changed bacterial phyla or genera were discovered between 14 and 21 d postpartum (*P* > 0.05).

### 3.3. Metabolic profiles of ruminal SCFAs in dairy cows within 21 d postpartum

**Figure 5:**
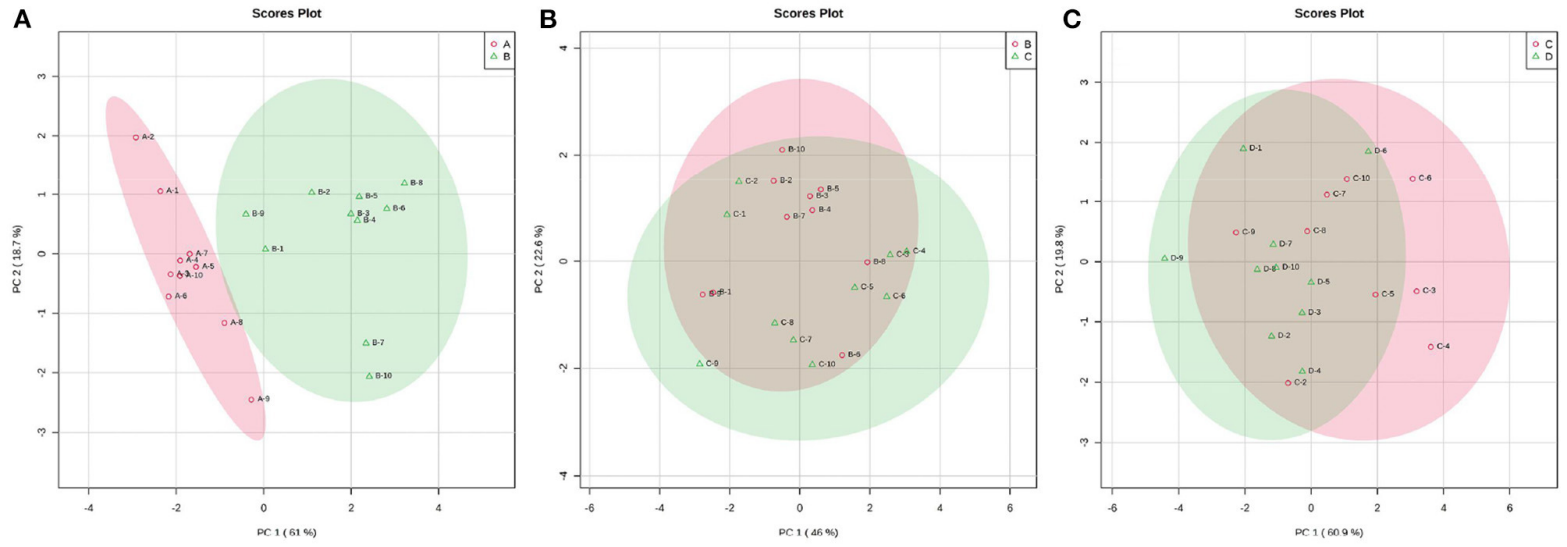
The 2D scatter plots of PCA of rumen SCFAs between groups A and B **(A)**, groups B and C **(B)**, and groups C and D **(C)**. The metabolic profiles of ruminal SCFAs were obviously waved within 7 d parturition and then gradually stabilized from 7 to 21 d postpartum.

Fluctuations in the metabolic profiles of ruminal SCFAs between the four groups were visualized with 2D scatter plots of PCA (Figure 5). The metabolic profiles between groups A and B were completely separated, but those between groups B and C and between groups C and D were largely merged. These fluctuations hinted that the rumen bacteria-producing SCFAs most likely occurred obvious alteration between 0 and 7 d postpartum and then gradually stabilized from 7 to 21 d postpartum.

**Figure 6:**
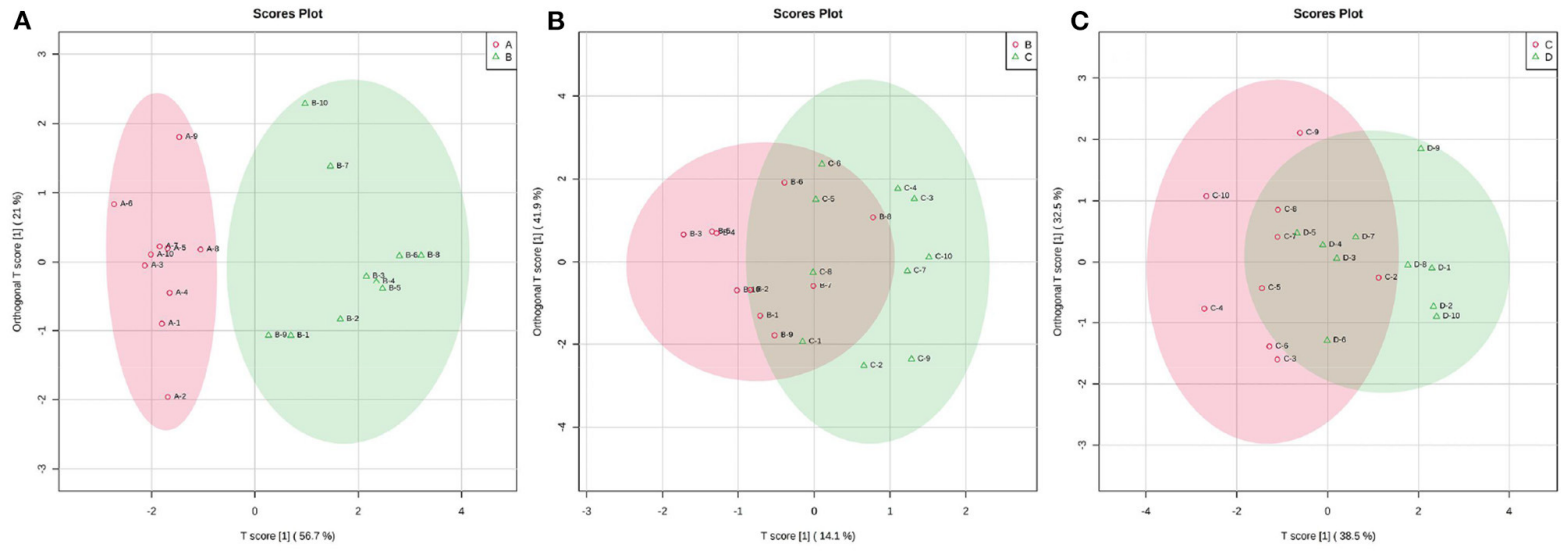
The 2D scatter plots of OPLS-DA of rumen SCFAs between groups A and B **(A)**, groups B and C **(B)**, and groups C and D **(C)**.

According to the developed linear regression equations (Supplementary Table 2), the concentrations of SCFAs in rumen were calculated (Table 1). A clear difference in the concentrations of SCFAs between 0 and 7 d postpartum was also observed via the models of OPLS-DA (Figure 6). Combining VIP values from OPLS-DA, *P*, and FC values from univariate analysis, the differential ruminal SCFAs between the four groups were ascertained according to the standard of VIP ≥ 1, *P* < 0.05, FC ≥ 2, or FC ≤ 0.5. The concentrations of propionic acid, butyric acid, and valeric acid were significantly higher at 7 d postpartum than those at 0 d postpartum, while the concentrations of acetic acid, isobutyric acid, isovaleric acid, and caproic acid were not distinctly changed from 0 to 7 d postpartum. There were no significant differences in the concentrations of SCFAs between 7 and 14 d and between 14 and 21 d.

**Table 1:** The concentrations (μg·mL-1) and the related parameters of SCFAs.

**Metabolites**	**Group A**	**Group B**	**A vs. B**			**Group C**	**B vs. C**			**Group D**	**C vs. D**		
	**Mean** ±**SD**		**FC** ^a^	**VIP** ^b^	***P-*** **Value**	**Mean** ±**SD**	**FC**	**VIP**	***P*** **-value**	**Mean** ±**SD**	**FC**	**VIP**	***P-*** **Value**
Acetic acid	634.00 ± 294.36	706.60 ± 261.78	1.12	1.30	0.59	1006.40 ± 256.23	1.42	1.73	0.02	1100.30 ± 272.47	1.09	1.35	0.46
Propionic acid	323.68 ± 165.61	660.50 ± 238.35	2.04 ↑	1.27	0.003^**^	781.60 ± 323.67	1.46	1.15	0.12	706.60 ± 204.17	0.90	1.32	0.59
Isobutyric acid	36.64 ± 23.23	32.16 ± 13.77	0.88	1.10	0.63	23.86 ± 7.67	1.49	1.02	0.05	26.90 ± 10.12	0.85	1.26	0.33
Butyric acid	328.17 ± 149.00	693.90 ± 243.97	2.11↑^c^	1.03	0.012^*^	31.56 ± 7.85	0.98	0.85	0.91	46.36 ± 13.60	1.47	1.15	0.01
Isovaleric acid	34.96 ± 20.46	26.04 ± 11.06	0.75	0.87	0.27	30.36 ± 16.91	1.32	0.82	0.18	43.65 ± 17.91	0.90	0.52	0.57
Valeric acid	41.68 ± 21.69	86.28 ± 32.71	2.07↑	1.81	0.004^**^	984.90 ± 392.93	1.05	0.46	0.83	832.40 ± 223.78	1.13	0.46	0.48
Caproic acid	14.17 ± 8.78	22.73 ± 13.63	1.60	0.08	0.13	126.00 ± 62.95	1.17	0.11	0.53	112.79 ± 32.97	1.44	0.26	0.12

^*a*^Fold change is the ratio of the relative peak intensity of the metabolites between the two groups (B/A, C/B, and D/C). ^*b*^Variable importance projection value is calculated from the orthogonal partial squares discriminant analysis model. ^*c*^↑ or ↓ shows the change trends of the differential metabolites in postpartum dairy cows (B/A, C/B, and D/C). The concentrations of propionic acid, butyric acid, and valeric acid were significantly higher within 7 d postpartum. ^*^ indicates *p* < 0.05, ^**^ indicates *p* < 0.01.

### 3.4. Metabolic profiles of other ruminal metabolites in dairy cows within 21 d postpartum

**Figure 7:**
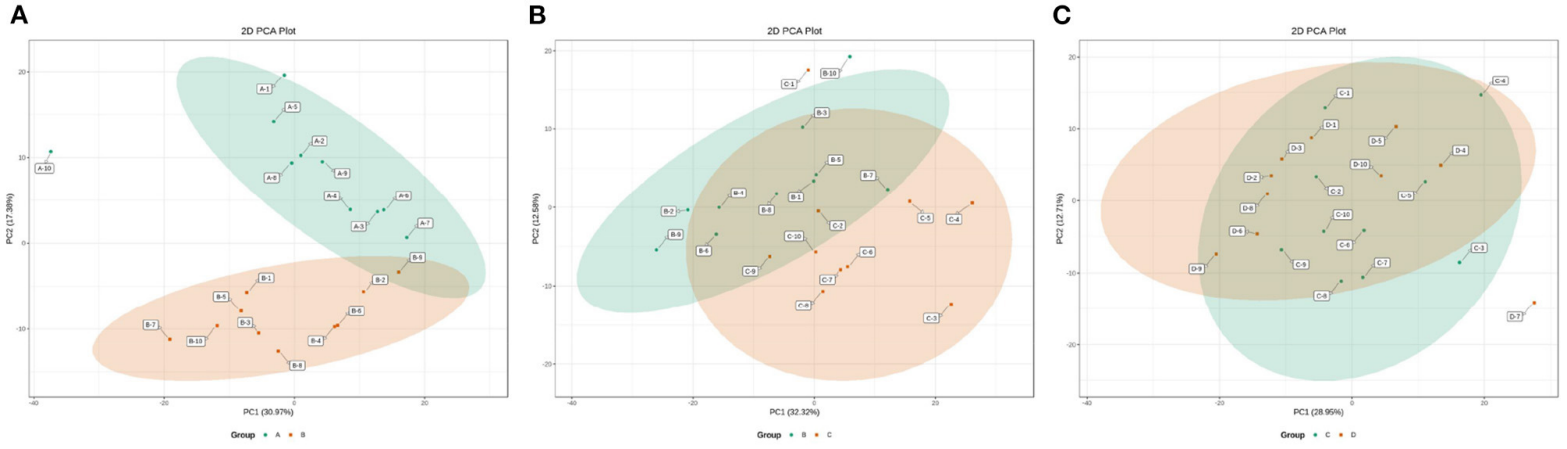
The 2D scatter plots of PCA of ruminal other metabolites between groups A and B **(A)**, groups B and C **(B)**, and groups C and D **(C)**.

The changes in the metabolic profile of other ruminal metabolites among the four groups were also visualized by the 2D scatter plots of PCA (Figure 7). In accordance with the ruminal SCFAs, there was a clear separation in metabolic profiles between groups A and B, while there were large overlaps in metabolic profiles between groups B and C and between groups C and D. The results indicated that the digestive ability of ruminal bacteria to feed occurred a sharp alteration from 0 to 7 d postpartum and then stabilized after 7 d postpartum.

**Figure 8:**
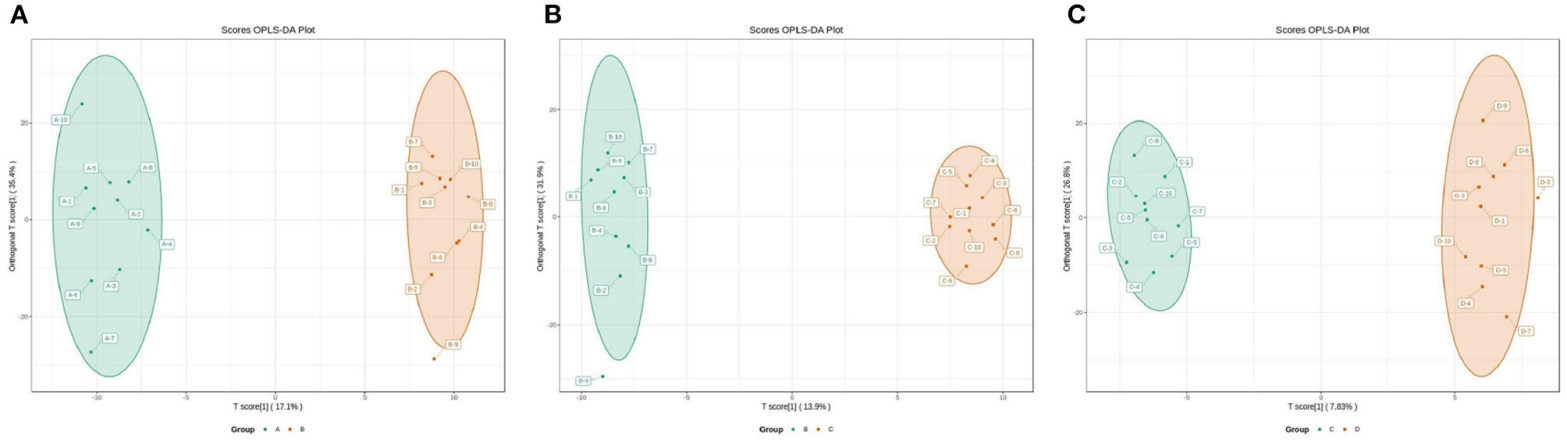
The 2D scatter plots of OPLS-DA of rumen other metabolites between groups A and B **(A)**, groups B and C **(B)**, and groups C and D **(C)**.

**Figure 9:**
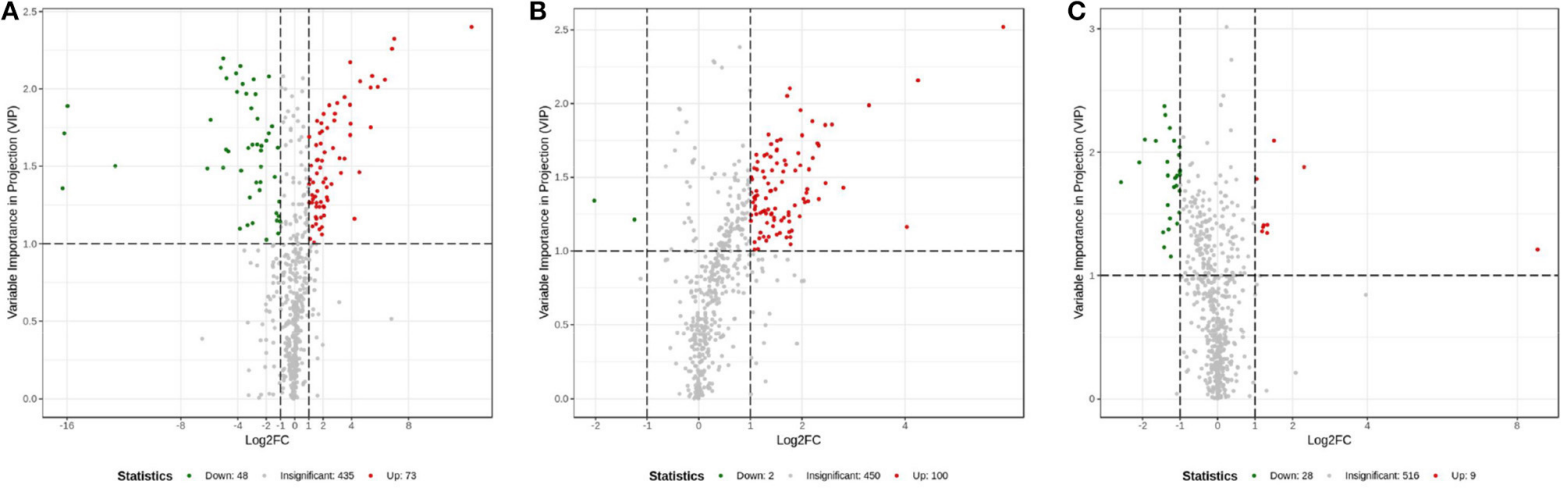
Volcanic plots of rumen other metabolites between groups A and B **(A)**, groups B and C **(B)**, and groups C and D **(C)**.

**Figure 10:**
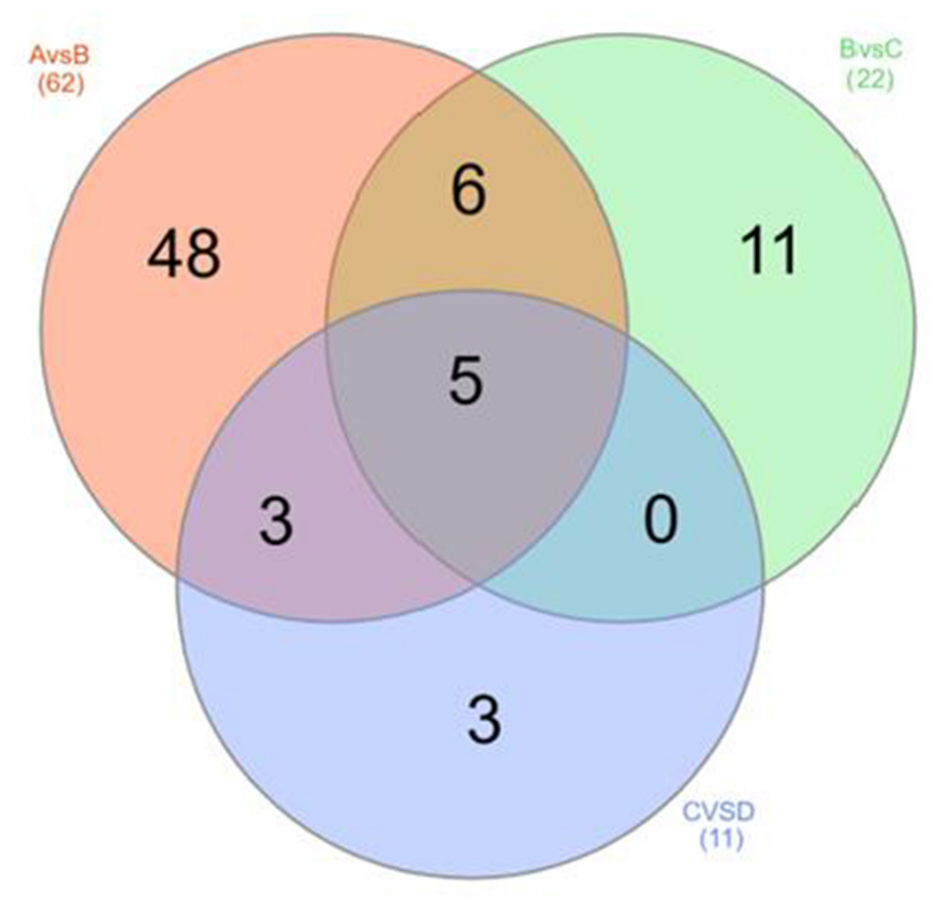
The Venn diagram of rumen differential metabolites between groups A and B, groups B and C, and groups C and D.

**Figure 11:**
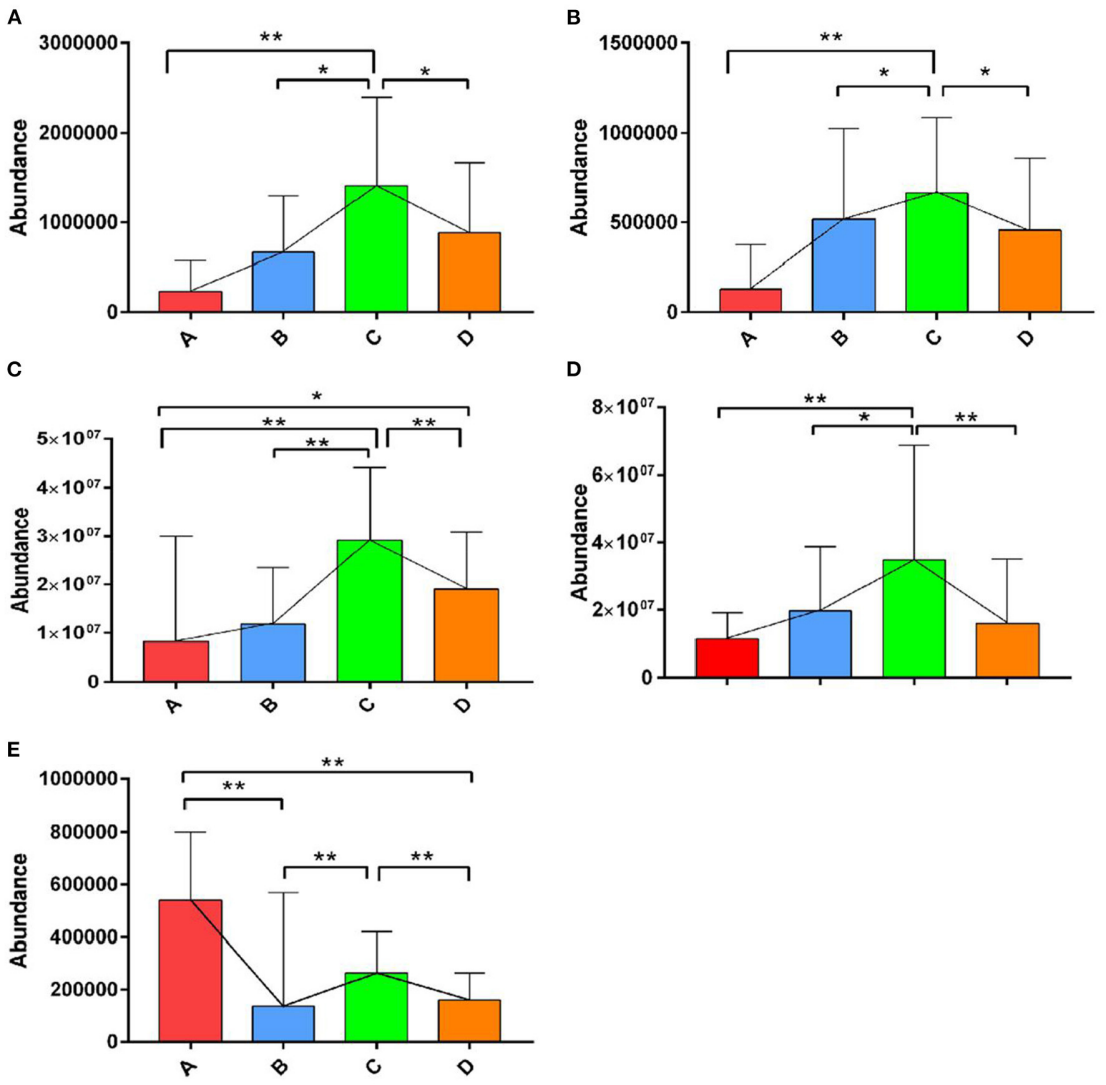
The relative abundances of the differential metabolites of D-glucose, lactose, tyramine, adenine, and pyridoxal in the four groups. ** indicates extremely significant difference (*p* < 0.01), and * indicates significant difference (*p* > 0.05). The levels of lactose **(A)**, D-glucose **(B)**, tyramine **(C)**, and adenine **(D)** in rumen were significantly increased from 0 to 14 d and decreased from 14 to 21 d postpartum. The level of pyridoxal **(E)** in rumen was evidently significantly higher at 0 d postpartum than that at 7, 14, and 21 d postpartum.

The OPLS-DA models were constructed to search the differential metabolites among the four groups (Figure 8). The OPLS-DA models presented the high discriminatory abilities to groups A and B (*R*^2^Y = 0.989, Q^2^ = 0.87), groups B and C (*R*^2^Y = 0.994, Q^2^ = 0.515), and groups C and D (*R*^2^Y = 0.989, Q^2^ = 0.442). *R*^2^Y close to 1 and Q^2^ higher than 0.4 indicates a good model fitting. According to the standard of VIP ≥ 1, *P* < 0.05, FC ≥ 2, or FC ≤ 0.5, the intergroup differential metabolites were confirmed (Figure 9; Supplementary Table 3). A total of 27 metabolites were obviously elevated and 35 metabolites were reduced from 0 to 7 d postpartum. In total, 18 metabolites were clearly increased and 4 metabolites decreased from 7 to 14 d postpartum. Overall, 3 metabolites distinctly ascended and 8 metabolites descended from 14 to 21 d postpartum. In total, 5 differential metabolites were shared between the 4 groups (Figure 10). The levels of lactose, D-glucose, tyramine, and adenine in the rumen were significantly increased from 0 to 14 d and decreased from 14 to 21 d postpartum. The level of pyridoxal in the rumen was evidently significantly higher at 0 d postpartum than that at 7, 14, and 21 d postpartum (Figure 11).

### 3.5. Correlation between ruminal bacteria and metabolites in dairy cows within 21 d postpartum

The correlation network diagrams between rumen bacteria and metabolites were generated using M^2^IA software (Supplementary Figure 2). At the phylum level, *Chloroflexi* was negatively correlated with propionic acid and valeric acid. *Euryarchaeota* was negatively associated with propionic acid. At the genus level, unidentified*_Prevotellaceae* showed a positive correlation with propionic acid, valeric acid, and butyric acid. *Flexilinea* was negatively correlated with propionic acid and valeric acid. *Methanobrevibacter* was negatively linked with propionic acid. Unidentified*_Ruminococcaceae* was positively associated with propionic acid, valeric acid, and butyric acid. Unidentified*_Christensenellaceae* was negatively associated with valeric acid, butyric acid, and propionic acid. *Pseudobutyrivibrio* showed a positive correlation with butyric acid. *Moryella* was positively associated with propionic acid and valeric acid. *Flexilinea* presented a positive association with pyridoxal. Unidentified*_Ruminococcaceae* was negatively correlated with pyridoxal and positively correlated with tyramine. *Methanobrevibacter* was positively linked with pyridoxal. The R- and *P*-values of bacteria with SCFAs and other metabolites are shown in Table 2.

**Table 2:** The correlation table between rumen bacteria and SCFAs and other metabolites in postpartum dairy cows.

**Bacteria**	**SCFAs and other metabolites**	***R***	***P***
**At phylum level**			
*Chloroflexi*	Propionic acid	−0.686	8.50E-04^**^
	Valeric acid	−0.614	4.00E-03^**^
*Euryarchaeota*	Propionic acid	−0.692	7.30E-04^**^
**At genus level**			
Unidentified_ *Prevotellaceae*	Propionic acid	0.651	1.90E-03^**^
	Valeric acid	0.52	0.019^*^
	Butyric acid	0.513	0.04^*^
*Flexilinea*	Propionic acid	−0.759	1.00E-04^**^
	Valeric acid	−0.598	5.30E-03^**^
*Methanobrevibacter*	Propionic acid	−0.669	1.30E-03^**^
Unidentified_ *Ruminococcaceae*	Propionic acid	0.657	1.60E-03^**^
	Valeric acid	0.591	6.10E-03^**^
	Butyric acid	0.556	0.011^*^
Unidentified_ *Christensenellaceae*	Propionic acid	−0.507	0.023^*^
	Valeric acid	−0.568	8.90E-03^**^
	Butyric acid	−0.695	6.80E-04^**^
*Pseudobutyrivibrio*	Butyric acid	0.511	0.021^*^
*Moryella*	Propionic acid	0.504	0.031^*^
	Valeric acid	0.518	0.033^*^
*Flexilinea*	Pyridoxal	0.481	0.032
*Methanobrevibacter*	Pyridoxal	0.459	0.042
Unidentified_ *Ruminococcaceae*	Pyridoxal	−0.567	9.10E-03
	Tyramine	0.720	3.40E-04

^*^ indicates *p* < 0.05, ^**^ indicates *p* < 0.01. |R| > 0.7 means a very tight correlation is very close, |R| between 0.4 and 0.7 means a tight correlation.

## 4. Discussion

### 4.1. Changes in ruminal bacteria and metabolic profiles in high-producing dairy cows within 21 d postpartum

The present study certified that the change trends in rumen bacteria were basically consistent with the metabolic profiles of ruminal SCFAs and other metabolites. ANOSIM analysis of beta diversity indicated that the structure of ruminal bacteria was sharply fluctuated within 14 d after parturition. 2D scatter plots of PCA displayed that the metabolic profiles of ruminal SCFAs and other metabolic profiles were obviously waved within 7 d parturition. In addition, some studies found that Firmicutes and Bacteroidetes phyla were rumen-dominant bacteria in healthy dairy cows during the late perinatal period (Difford et al., 2018; Mingyuan et al., 2018; Wang et al., 2019), which is consistent with the results of the present study. Our study further testified that the abundances of *Firmicutes* and *Bacteroidetes* held at steady level in the rumen of high-producing dairy cows within 21 d postpartum. However, the abundances of Euryarchaeota and Chloroflexi phyla were distinctly descended within 7 d postpartum. *Chloroflexi* plays a key role in methane production (Bovio et al., 2019). *Euryarchaeota*, also known as methanogenic bacteria, participates in the methanogenesis and degradation of other hydrocarbons (Baker et al., 2020). *Methanobrevibacter* belonging to *Euryarchaeota* is an important component of intestinal and rumen methanogenic archaea and is related to greater methane emission (Tapio et al., 2017). The abundance of the *Methanobrevibacter* genus was also obviously decreased within 7 d postpartum in this study. The production of methane in the rumen of dairy cows indicates loss of energy (Appuhamy et al., 2016; BetancurMurillo et al., 2022). High-producing dairy cows are prone to the metabolic status of NEB during the late perinatal period due to lactation initiation and lower DMI intake. Hence, the self-adaptive reduction in the abundance of rumen of *Euryarchaeota, Chloroflexi*, and *Methanobrevibacter* is beneficial to alleviate the metabolic stress of negative energy balance in postpartum high-producing dairy cows.

### 4.2. Correlation between ruminal bacteria and SCFAs in high-producing dairy cows within 21 d postpartum

SCFAs are the main products of feed fermented by ruminal bacteria; we found that the concentrations of ruminal propionic acid, butyric acid, and valeric acid were notably ascended within 7 d postpartum. As the important substrate for gluconeogenesis, propionic acid provides 40–70% of glucose (DeFrain et al., 2005), inhibits inflammation, and improves the immunity of the body (Walkenhorst et al., 2020). Butyric acid can regulate energy metabolism (Fukumori et al., 2022), inhibit the production of pro-inflammatory mediators stimulated by LPS and cytokines, and promote the release of anti-inflammatory cytokine IL-10 (Renato et al., 2011; Chang et al., 2014).

In this study, the relative abundances of unidentified_*Prevotellaceae* and *Pseudobutyrivibrio* were significantly increased and that of *Methanobrevibacter* decreased within 7 d postpartum. At the phylum level, *Bacteroidetes* and *Firmicutes* were the ruminal dominant bacteria in dairy cows within 21 d postpartum. Unidentified_*Prevotellaceae* of Bacteroidetes phylum is one of the most abundant bacterial genera in the rumen, accounting for 45–60% of the total bacterial population (Jiang et al., 2017). Unidentified_*Prevotellaceae* can decompose starch and protein (Xie et al., 2019) and synthesize propionate, butyrate, and valerate (Salonen et al., 2014; Baothman et al., 2016). *Pseudobutyrivibrio* genus of *Firmicutes* is an effective bacterium degrading hemicellulose, which can produce butyrate (Louis and Flint, 2017). *Methanobrevibacter* genus of *Euryarchaeota* phylum can utilize large amount of propionic acid during methane production (Shi et al., 2014; Poehlein et al., 2018).

Additionally, the study verified that the relative abundance of unidentified_*Ruminococcaceae* was significantly increased at 7 d postpartum and then evidently decreased at 14 d postpartum and that of unidentified_*Christensenellaceae* was distinctly reduced within 14 d postpartum. Unidentified_*Ruminococcaceae*, the main ruminal cellulose-degrading bacteria, can produce butyrate and valerate, participating in the release of inflammatory and cytotoxic factors, immune regulation, and intestinal homeostasis (Fanli et al., 2016; Daniela et al., 2019). *Christensenellaceae* have been reported to produce volatile fatty acids by utilizing a variety of sugars (Morotomi et al., 2012). Consequently, the levels of SCFAs in the rumen were closely linked to the abundance of SCFAs-producing and utilizing bacteria (Wang et al., 2021).

Hence, the correlation analysis was performed in this study to further validate the relationship between rumen bacteria and SCFAs. The results showed that unidentified_*Prevotellaceae* positively correlated with propionic acid, butyric acid, and valeric acid; *Methanobrevibacter* was negatively associated with propionic acid; unidentified_*Ruminococcaceae* was positively related to butyric acid, propionic acid, and valeric acid; unidentified_*Christensenellaceae* was negatively linked with butyric acid; and *Pseudobutyrivibrio* was positively correlated with butyric acid. In summary, these changes in the abundance of ruminal SCFAs-producing and utilizing bacteria may contribute to the high-producing dairy cows coping with the challenge of postpartum metabolic stress and inflammatory response.

### 4.3. Correlation between rumen bacteria and other metabolites in high-producing dairy cows within 21 d postpartum

In this study, the level of pyridoxal, the main component of vitamin B6, significantly declined 7 d postpartum and then clearly elevated within 14 d postpartum. The correlation analysis further certified that pyridoxal was negatively correlated with unidentified_*Ruminococcaceae* and positively associated with *Methanobrevibacter*. Rumen bacteria can synthesize B vitamins (Zinn et al., 1987). The synthesis of vitamin B6 in the rumen is negatively correlated with the abundance of fiber-degrading bacteria such as unidentified_*Ruminococcaceae* (Castagnino et al., 2016), which is consistent with the results of the present study. *Methanobrevibacter* facilitates the biosynthesis of most B vitamins in the small intestine (Jiang et al., 2022). However, it has not been reported whether *Methanobrevibacter* can promote the synthesis of B vitamins in rumen but its effect on the rumen has not been reported. Our study indicated that *Methanobrevibacter* in the rumen might aid the biosynthesis of B vitamin.

A highly significant positive correlation between tyramine content and unidentified_*Ruminococcaceae* was attested in the study. Biogenic amines are produced through the decarboxylation of some amino acids such as tyrosine and histidine under the action of bacterial amino acid decarboxylase (Aschenbach and Gäbel, 2000). The production of biogenic amines is closely related to the rumen bacteria (Phuntsok et al., 1998). *Ruminococcus gnavus* of Firmicutes phylum mediate the catabolism of phenylalanine, thus promoting the production of tyramine (Wu et al., 2023; Zhai et al., 2023). *Ruminococcus gnavus* is linked with tyramine generation (Yali et al., 2020).

Adenine is the precursor of ruminal microbial crude protein (MCP). MCP is generated *via* rumen microorganisms-fermenting feed in dairy cows (Lu et al., 2019). The efficiency of MCP synthesis in rumen ascends with the increase of DMI in dairy cows (Abdukarim, 2019). Protozoa synthesize their own nucleic acids utilizing free adenine and urine through the remedial pathway in the rumen (McAllan, 1982). We found that the level of adenine in the rumen was obviously elevated with 14 d postpartum and then clearly dropped, and there was no correlation between adenine and bacteria. The results suggest that the level of adenine is most likely related to the relative abundance of protozoa in the rumen.

As the main starch-degrading bacteria in the rumen, unidentified_*Prevotellaceae* generates amylases that hydrolyze straight-chain or branched-chain starches into glucose and lactose through the pathway of sucrose, galactose, and starch metabolism (Richard et al., 2003). However, the soluble sugars are rapidly converted into volatile fatty acids (VFAs) in the rumen as the source of energy for organisms (Lucy et al., 2013). In this study, the change trends of lactose and D-glucose were found similar to those of adenine within 21 d postpartum, and there were no correlations between the two and unidentified_*Prevotellaceae* and other ruminal bacteria. The uncorrelation was highly possibly attributed to the rapid transformation of lactose into D-glucose.

## 5. Conclusion

To reveal the dynamic changes of rumen bacteria and metabolites in high-producing dairy cows after parturition, 16S rRNA high-throughput sequencing, GC-MS/MS targeted metabolomics, and UPLC-MS/MS untargeted metabolomics were used in this study, to comprehensively investigate the changes of ruminal bacterial abundance, SCFAs, and other metabolites in high-producing dairy cows at 0, 7, 14, and 21 d postpartum and the correlation between the three. The results suggested that rumen bacteria and SCFAs and other metabolites took place various degrees of fluctuations during the late perinatal period affected by parturition stress and lactation initiation and that the levels of ruminal propionic acid, butyric acid, valeric acid, and pyridoxal presented obvious correlation with the Chloroflexi and Euryarchaeota phyla, as well as the *Prevotellacea, Flexilinea, Ruminococcaceae, Christensenellaceae, Moryella Pseudobutyrivibrio, and Methanobrevibacter* genera. The results would provide some references for the nutrition management and prevention of metabolic disease in postpartum high-producing dairy cows. The subsequent experiments should focus on the dynamic changes in rumen protozoa, archaea, and fungi and correlations with metabolites in postpartum high-producing dairy cows.

## Data availability statement

The datasets presented in this study can be found in online repositories. The names of the repository/repositories and accession number(s) can be found below: 16s rRNA NCBI-PRJNA1000975, UPLC-MS/MS EBI-MTBLS8333.

## Ethics statement

The animal study was approved by the Institutional Animal Care and Use Committee of Ningxia University (NXUC20200618). The study was conducted in accordance with the local legislation and institutional requirements.

## Author contributions

YoM: Data curation, Formal analysis, Methodology, Writing—original draft, Writing—review and editing. FW: Conceptualization, Formal analysis, Writing—review and editing. WK: Conceptualization, Methodology, Writing—review and editing. RW: Methodology, Resources, Writing—review and editing. XL: Methodology, Writing—review and editing. HD: Methodology, Writing—review and editing. YuM: Conceptualization, Formal analysis, Writing—review and editing. YG: Conceptualization, Supervision, Writing—review and editing.
