# Supplementary material for: Dynamic changes of rumen bacteria and their fermentative ability in high-producing dairy cows during the late perinatal period
Source: Front Microbiol. 2023 Sep 25;14:1269123. doi: 10.3389/fmicb.2023.1269123 (PMC10560760; doi:10.3389/fmicb.2023.1269123)
Supplement: Supplementary file 1 [file Data_Sheet_1.docx]

Supplementary Material

Dynamic changes of rumen bacteria and their fermentive ability in high-producing dairy cows during late perinatal period

Yongxia Mao ^1^, Feifei Wang^1^, Weiyi Kong ^1^, Ruiling Wang ^1^, Xin Liu^1^, Hui Ding ^1^, Yansheng Guo *

*** Correspondence:** Yansheng Guo: [guoyansheng1978@163.com](mailto:guoyansheng1978@163.com)

# Supplementary Tables

**Supplemental Table S1.** The TMR diet formula of dairy cows after parturition

| Composition | Content（%） | Item | Unit（%） |
| --- | --- | --- | --- |
| Whole corn silage | 34.48 | Crude Protein | 17.51 |
| Corn - Fine | 12.43 | Adj Tot Starch | 22.80 |
| Soybean Hulls | 8.35 | NDF | 28.09 |
| Soybean Meal-Protein | 5.16 | Fat | 2.71 |
| Molasses-Cane | 2.82 | NEL (MJ·kg^-1^) | 2.47 |
| Cottonseed Meal | 6.89 |  |  |
| Soybeans - Whole | 1.62 |  |  |
| Fat - Energy | 0.75 |  |  |
| AA-MetaSmart | 0.09 |  |  |
| Alfalfa Hay | 11.82 |  |  |
| Oat Hay-oot | 4.85 |  |  |
| Premix^1^ | 3.87 |  |  |
| Corn Hulls | 1.96 |  |  |
| DDGS | 2.49 |  |  |
| Sodium Bicarbonate | 0.36 |  |  |
| Magnesium Oxide54% | 0.12 |  |  |
| Salt | 0.25 |  |  |
| Calcium carbonate | 0.82 |  |  |
| UREA | 0.38 |  |  |
| Yeast culture | 0.49 |  |  |
| total | 100 |  |  |

Note: Per kilogram of premix contains the following: VA 650 KIU, VD 3300 KIU, VE 9000 IU, D-biotin 90 mg, Nicotinic acid 30000 mg, Zn5000 mg, Cu 1700 mg, Mn 4000 mg, I 100 mg, Se 50 mg, Co 52 mg.

**Supplemental Table S2.** The linear regression equation, correlation coefficient and linear range of SCFAs

| Component | Retention Time (min) | Equation | R | LLOQ~ULOQ  (ng/ml) |
| --- | --- | --- | --- | --- |
| Acetic acid | 3.36 | y = 0.642 * x + 0.027 | 0.994 | 0.36~360 |
| Propionic acid | 3.97 | y = 0.460 * x + 0.001 | 0.998 | 0.36~171 |
| Isobutyric acid | 4.14 | y = 1.002 * x - 0.002 | 0.998 | 0.09~360 |
| Butyric acid | 4.55 | y = 5.565 * x + 0.200 | 0.999 | 0.36~360 |
| Isovaleric acid | 4.80 | y = 6.493 * x - 0.001 | 0.100 | 0.09~360 |
| Valeric acid | 5.22 | y = 7.121* x + 0.127 | 0.100 | 0.09~360 |
| Caproic acid | 5.79 | y = 3.386 * x + 0.231 | 0.999 | 3.60~360 |

**Supplemental Table S3.** The differential metabolites and related Parameters of high-producing dairy cows after delivery (A vs B, B vs C, C vs D)

| Differential metabolites | FC | VIP | *P* value | FALSE | Type |
| --- | --- | --- | --- | --- | --- |
| A vs B |  |  |  |  |  |
| Thiamine | 19.51 | 1.21 | 2.22E-02 | 8.41E-02 | up |
| Lactose | 11.66 | 1.60 | 1.11E-03 | 8.44E-03 | up |
| O-Succinyl-L-Homoserine | 9.65 | 1.82 | 7.66E-05 | 9.38E-04 | up |
| Guanine | 7.41 | 1.46 | 4.37E-02 | 6.20E-02 | up |
| D-Glucose | 5.63 | 1.51 | 3.05E-03 | 1.66E-02 | up |
| Maltose | 5.92 | 1.32 | 1.07E-02 | 5.26E-02 | up |
| 5-Oxoproline | 4.77 | 1.97 | 6.11E-06 | 1.39E-04 | up |
| Pyridoxine | 4.41 | 1.32 | 1.10E-02 | 5.26E-02 | up |
| Spermidine | 3.67 | 1.83 | 6.50E-05 | 8.77E-04 | up |
| Adipic Acid | 3.02 | 1.69 | 4.28E-04 | 3.80E-03 | up |
| Pyridoxine 5'-Phosphate | 2.09 | 1.62 | 8.91E-04 | 7.21E-03 | up |
| D-Alanyl-D-Alanine | 2.47 | 1.48 | 3.34E-03 | 2.06E-02 | up |
| Tyramine | 2.5 | 1.60 | 1.11E-03 | 8.44E-03 | up |
| N-Methyl-L-Glutamate | 2.97 | 1.88 | 3.09E-05 | 4.88E-04 | up |
| 2-Methylsuccinic Acid | 2.28 | 1.73 | 2.58E-04 | 2.69E-03 | up |
| 4-Pyridoxic Acid | 2.22 | 1.73 | 2.71E-04 | 2.74E-03 | up |
| Glutaric Acid | 2.28 | 1.72 | 2.97E-04 | 2.89E-03 | up |
| Epinephrine | 2.22 | 1.72 | 3.02E-04 | 2.89E-03 | up |
| L-Cystine | 2.74 | 1.08 | 4.44E-02 | 1.42E-01 | up |
| Nicotinic Acid | 2.27 | 1.81 | 8.81E-05 | 1.03E-03 | up |
| 2-Picolinic Acid | 2.59 | 1.81 | 1.00E-04 | 1.14E-03 | up |
| Thymidine | 2.00 | 1.24 | 1.80E-02 | 7.30E-02 | up |
| Deoxycytidine | 2.19 | 1.21 | 2.21E-02 | 8.41E-02 | up |
| Uridine | 2.18 | 1.16 | 2.86E-02 | 1.03E-01 | up |
| Riboflavin | 2.11 | 1.34 | 9.76E-03 | 5.08E-02 | up |
| L-Ornithine | 2.86 | 1.28 | 1.43E-02 | 6.28E-02 | up |
| Adenine | 2.16 | 1.31 | 1.17E-02 | 5.54E-02 | up |
| Pyridoxal | 0.31 | 1.91 | 1.83E-05 | 3.50E-04 | down |
| L-Lactic Acid | 0.41 | 1..62 | 4.38E-02 | 3.60E-03 | down |
| L-Alanine | 0.49 | 1.01 | 4.73E-02 | 7.73E-02 | down |
| N-Acetyl-L-Tyrosine | 0.15 | 1.34 | 9.71E-03 | 5.08E-02 | down |
| Biotin | 0.09 | 2.19 | 6.47E-09 | 1.18E-06 | down |
| D-Xylulose 5-phosphate | 0.09 | 1.86 | 4.39E-05 | 6.66E-04 | down |
| 3'-Aenylic Acid | 0.09 | 2.12 | 1.11E-07 | 6.75E-06 | down |
| Uridine 5-Monophosphate | 0.07 | 2.11 | 1.48E-07 | 7.69E-06 | down |
| Cytidine-5-Monophosphate | 0.08 | 2.11 | 1.84E-07 | 8.39E-06 | down |
| Uric Acid | 0.19 | 2.04 | 1.35E-06 | 4.91E-05 | down |
| Adenosine | 0.16 | 2.03 | 1.62E-06 | 5.37E-05 | down |
| 2'-Deoxyadenosine-5'-Monophosphate | 0.05 | 2.02 | 2.19E-06 | 6.13E-05 | down |
| N-Acetyl-L-alanine | 0.02 | 1.90 | 2.36E-05 | 4.10E-04 | down |
| Ribulose-5-Phosphate | 0.21 | 1.74 | 2.50E-04 | 2.68E-03 | down |
| 2'-Deoxycytidine-5'-Monophosphate | 0.23 | 1.66 | 6.31E-04 | 5.47E-03 | down |
| Creatinine | 0.02 | 1.59 | 1.29E-03 | 9.61E-03 | down |
| Lysopc 14:0 | 0.19 | 1.39 | 3.65E-03 | 8.56E-03 | down |
| Lysopc 15:0 | 0.27 | 1.82 | 7.73E-05 | 9.38E-04 | down |
| Lysopc 16:1 | 0.28 | 1.24 | 1.76E-02 | 7.21E-02 | down |
| Lysopc 18:1 | 0.23 | 1.56 | 1.60E-03 | 1.14E-02 | down |
| Lysopc 18:2 | 0.32 | 1.54 | 1.92E-03 | 1.32E-02 | down |
| 2-Aminoethanesulfonic Acid | 0.17 | 1.54 | 2.01E-03 | 1.35E-02 | down |
| 2-Deoxyribose 1-Phosphate | 0.37 | 1.51 | 2.59E-03 | 1.69E-02 | down |
| Creatine | 0.03 | 1.5 | 2.81E-03 | 1.76E-02 | down |
| N-Methyl-D-Aspartic Acid | 0.47 | 1.41 | 5.88E-03 | 3.35E-02 | down |
| L-Proline | 0.43 | 1.21 | 2.15E-02 | 8.41E-02 | down |
| L-Aspartic Acid | 0.45 | 1.17 | 2.17E-02 | 8.41E-02 | down |
| 1-Methylxanthine | 0.07 | 1.2 | 3.26E-02 | 1.14E-01 | down |
| N-Acetyl-L-Leucine | 0.2 | 1.13 | 3.70E-02 | 7.30E-02 | down |
| Succinic Acid | 0.43 | 1.02 | 4.30E-03 | 9.78E-02 | down |
| Aminomalonic Acid | 0.42 | 1.02 | 2.50E-02 | 1.15E-01 | down |
| N-Acetylmethionine | 0.46 | 0.94 | 2.90E-02 | 9.97E-02 | down |
| Methylmalonic Acid | 0.44 | 0.93 | 9.00E-03 | 9.93E-02 | down |
| Nalpha-Acetyl-L-glutamine | 0.34 | 0.98 | 1.30E-02 | 9.97E-02 | down |
| Glycochenodeoxycholic Acid | 0.25 | 1.67 | 6.50E-03 | 1.47E-01 | down |
| B vs C |  |  |  |  |  |
| Citric Acid | 16.37 | 1.16 | 3.00E-03 | 1.57E-01 | up |
| Succinic Acid | 5.00 | 1.72 | 5.00E-03 | 1.44E-01 | up |
| L-Lactic Acid | 4.60 | 1.88 | 2.00E-03 | 9.57E-02 | up |
| 2-Hydroxybutanoic Acid | 3.31 | 1.83 | 1.48E-03 | 9.38E-02 | up |
| Adenine | 2.42 | 1.54 | 3.90E-02 | 1.57E-01 | up |
| N-Acetylneuraminic Acid | 3.53 | 1.71 | 3.74E-03 | 9.94E-02 | up |
| Pyridoxal | 3.37 | 1.20 | 6.00E-03 | 1.44E-01 | up |
| Lysope 14:0 | 3.17 | 1.61 | 5.00E-03 | 9.95E-02 | up |
| D-Glucarate | 3.50 | 1.49 | 1.41E-02 | 1.35E-01 | up |
| Guanosine | 2.75 | 1.23 | 4.10E-02 | 9.94E-02 | up |
| D-Glucose | 2.51 | 1.01 | 2.00E-03 | 9.47E-02 | up |
| 3-Hydroxybutyrate | 2.41 | 1.65 | 1.50E-02 | 1.85E-01 | up |
| Pyridoxine 5'-Phosphate | 2.50 | 1.66 | 7.00E-03 | 9.95E-02 | up |
| Lactose | 2.37 | 1.27 | 1.80E-02 | 1.54E-01 | up |
| Tyramine | 2.47 | 1.36 | 3.70E-02 | 1.37E-01 | up |
| Adenosine | 2.10 | 1.56 | 1.40E-02 | 1.26E-01 | up |
| N-Acetyl-L-Glutamic Acid | 2.36 | 1.61 | 7.14E-03 | 1.13E-01 | up |
| L-Alanine | 2.04 | 1.48 | 1.49E-02 | 1.36E-01 | up |
| Creatine | 0.25 | 1.34 | 6.00E-03 | 1.26E-01 | down |
| Menaquinone | 0.48 | 1.72 | 3.48E-03 | 9.94E-02 | down |
| Thiamine Triphosphate | 0.45 | 1.34 | 4.67E-02 | 1.84E-01 | down |
| 3-Hydroxyphenylacetate | 0.48 | 1.38 | 2.54E-02 | 1.57E-01 | down |
| C vs D |  |  |  |  |  |
| 2'-Deoxyadenosine-5'-Monophosphate | 6.48 | 1.86 | 2.94E-02 | 5.52E-01 | up |
| Lysopc 15:0 | 2.36 | 1.55 | 3.66E-02 | 5.52E-01 | up |
| D-Xylulose 5-phosphate | 3.38 | 1.51 | 1.30E-02 | 9.63E-02 | up |
| Adenine | 0.44 | 1.97 | 1.99E-02 | 5.18E-01 | down |
| gamma-Aminobutyric Acid | 0.45 | 1.72 | 4.98E-02 | 5.52E-01 | down |
| Thymidine | 0.47 | 1.73 | 3.50E-02 | 1.57E-01 | down |
| Lactose | 0.46 | 1.85 | 4.63E-02 | 7.65E-02 | down |
| D-Glucose | 0.45 | 1.62 | 2.76E-02 | 3.79E-01 | down |
| Tyramine | 0.49 | 2.08 | 4.76E-02 | 6.56E-02 | down |
| Cytosine | 0.41 | 2.19 | 9.00E-03 | 3.59E-01 | down |
| Pyridoxal | 0.45 | 1.98 | 2.00E-03 | 2.73E-01 | down |

# Supplementary Figures

**Supplemental Figure S1:**

**
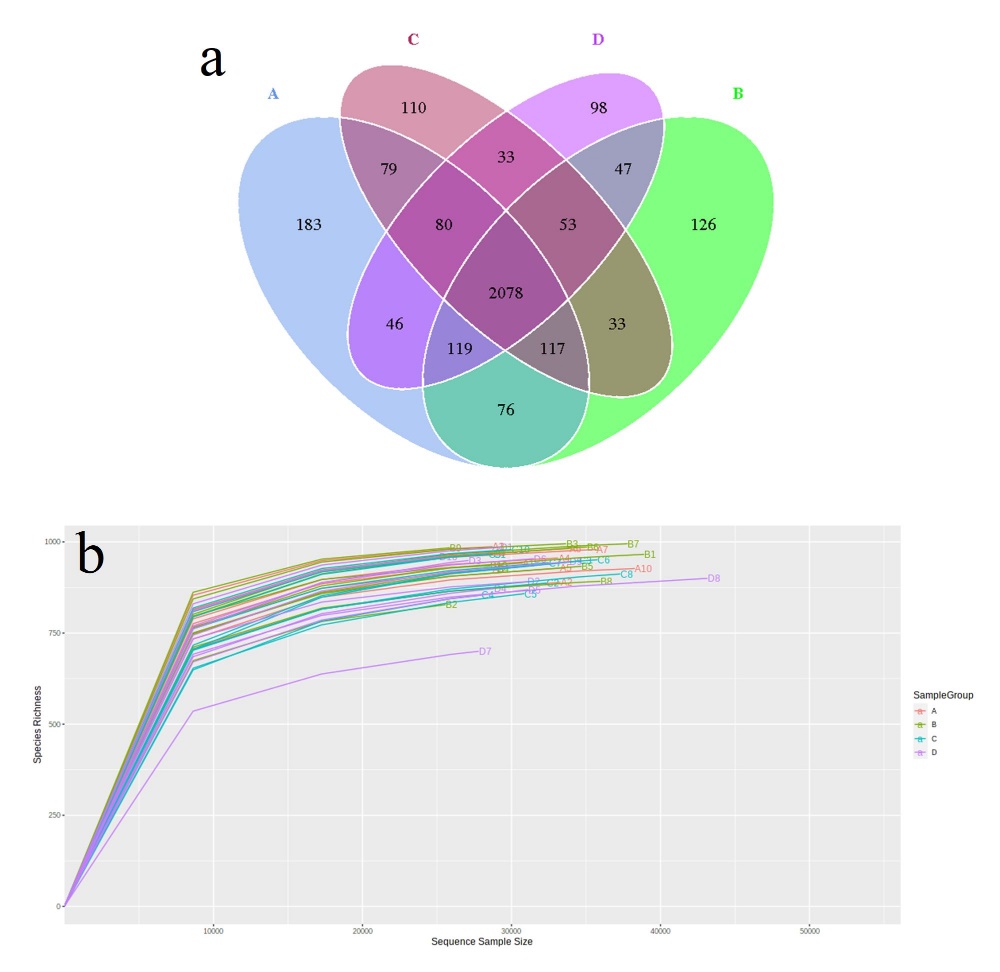
**

Supplemental Figure S1: The venn diagram (a) of OTUs and rarefaction curve (b) of four groups of rumen fluids in dairy cows within 21 d after calving

**Supplemental Figure S2:**


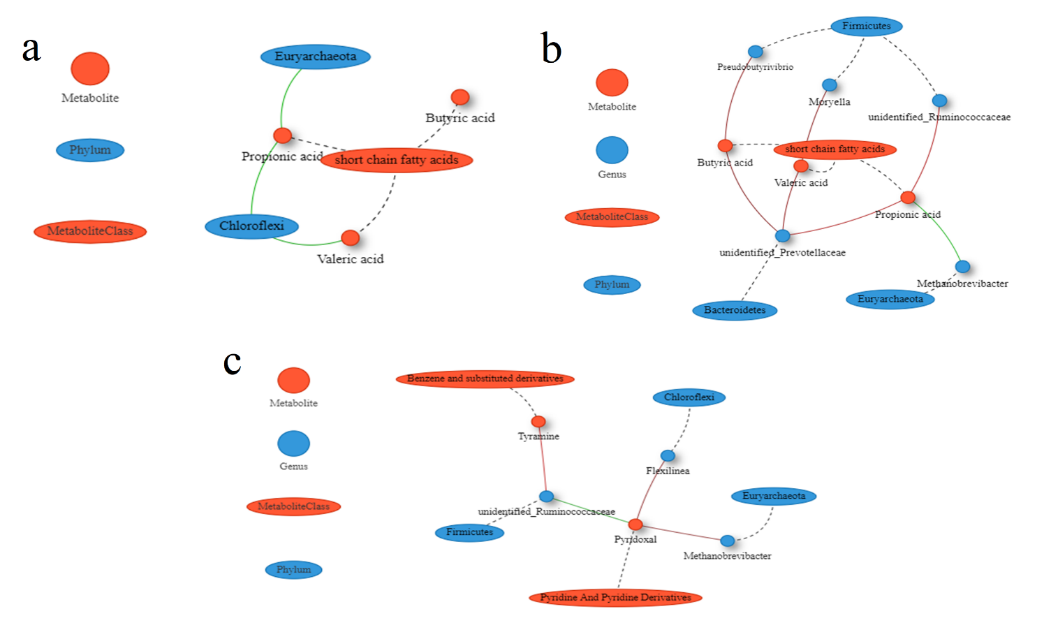


Supplemental Figure S2: Correlation network plots between rumen bacteria and SCFAs at phylum (a) and genus (b) levels and between rumen bacteria and other metabolites at genus level (c)

Note: The red line and green lines represent positive correlation and negative correlation respectively, and the dotted lines indicate affiliation. Metabolites are represented by red circles, microorganisms at phylum level are represented by blue ellipses, and microorganisms at genus level by blue circles. The red ellipse is the general name of short chain fatty acids.
